# CaMKII Splice Variants in Vascular Smooth Muscle Cells: The Next Step or Redundancy?

**DOI:** 10.3390/ijms23147916

**Published:** 2022-07-18

**Authors:** Finn T. Roberts-Craig, Luke P. Worthington, Samuel P. O’Hara, Jeffrey R. Erickson, Alison K. Heather, Zoe Ashley

**Affiliations:** 1 Department of Medicine, University of Otago, Dunedin 9016, New Zealand; robfi355@student.otago.ac.nz; 2 Department of Physiology, School of Biomedical Sciences, University of Otago, Dunedin 9016, New Zealand; luke.worthington@otago.ac.nz (L.P.W.); sam.ohara@otago.ac.nz (S.P.O.); jeff.erickson@otago.ac.nz (J.R.E.); alison.heather@otago.ac.nz (A.K.H.); 3 HeartOtago, University of Otago, Dunedin 9016, New Zealand

**Keywords:** CaMKII delta, CaMKII gamma, vascular

## Abstract

Vascular smooth muscle cells (VSMCs) help to maintain the normal physiological contractility of arterial vessels to control blood pressure; they can also contribute to vascular disease such as atherosclerosis. Ca^2+^/calmodulin-dependent kinase II (CaMKII), a multifunctional enzyme with four isoforms and multiple alternative splice variants, contributes to numerous functions within VSMCs. The role of these isoforms has been widely studied across numerous tissue types; however, their functions are still largely unknown within the vasculature. Even more understudied is the role of the different splice variants of each isoform in such signaling pathways. This review evaluates the role of the different CaMKII splice variants in vascular pathological and physiological mechanisms, aiming to show the need for more research to highlight both the deleterious and protective functions of the various splice variants.

## 1. Introduction

Ca^2+^/calmodulin-dependent kinase II (CaMKII) contributes to both physiological and pathological signaling pathways within vascular smooth muscle cells (VSMCs) (reviewed in [1]). Current research suggests the opposing roles of the CaMKII δ and γ isoforms within VSMCs [2]. CaMKIIδ is linked to worsening neointimal hyperplasia through CaMKII-mediated VSMC proliferation and migration [3,4]. In contrast, CaMKIIγ expression is predominant in physiological functions such as VSMC contractility, and its downregulation is related to VSMC phenotypic switching seen in vascular disease states [2,5,6]. The role of the different CaMKII isoforms is becoming well established in the literature; however, such evidence has been derived from total and isoform-specific knockdown models, despite well documented alterative splicing events across the different isoforms [7,8]. As a result, little is known about the specific protective or deleterious roles of individual splice variants. CaMKII isoforms are differentially expressed across tissue types in the body; thus, splice-variant-specific knockdown models may provide a novel therapeutic mechanism to protect against vascular pathologies. The aim of this review is to reflect and build on the current understanding of specific isoforms and splice variants in CaMKII-mediated vascular pathologies.

## 2. Structure, Activation and Diversity of CaMKII Gene Products

First coined in 1983, CaMKII is a serine/threonine kinase formed through the oligomerization of 6 to 12 monomer subunits [9,10]. Each subunit has three domains—the catalytic (N-terminal), regulatory, and association (C-terminal) domains [11]. These domains maintain the enzymatic action of the kinase, regulate the functions of the catalytic domain, and promote the assembly of oligomers, respectively [10,12,13]. 

Since their initial discovery, the four isoforms of CaMKII have been identified (α, β, δ, and γ), each encoded by a different gene (*Camk2a*, *Camk2b*, *Camk2d*, and *Camk2g*) [6]. The α and β isoforms were the first to be identified—being restricted largely to the brain and neural tissue—while the δ and γ isoforms have been identified within a variety of other tissues, including both the heart and vasculature [14]. The four isoforms remain relatively homologous in structure, with largely conserved catalytic and association domains; diversity in gene product function is produced by alternative splicing within the variable domain, found between the catalytic and association domains [7,15,16].

As there are numerous isoforms and splice variants, the oligomerization of subunits forms a heterooligomeric holoenzyme with varying levels of each subunit in the overall structure [9,17]. The alternative splicing of isoforms determines specific functional differences between variants; the ratio of each splice variant within a given holoenzyme, thus, can be considered deterministic of its specific functionality [7,17]. Ubiquitous CaMKII or isoform-specific knockout and overexpression studies are a limitation to expanding our understanding of the kinase’s function within the vasculature, as such experiments negate the role of specific isoforms and splice variants in cell localization, enzyme function, activity, and activation [7]. The complex tertiary structure of CaMKII and its subsequent multifunctional role allows for advanced signaling; the kinase is able to detect Ca^2+^ oscillations and integrate them to produce intracellular responses in the presence of changing CaM and Ca^2+^ concentrations [18]. 

While autophosphorylation seems to be the key activation mechanism conserved across all isoforms, numerous alternative mechanisms of activation have been proposed and established, typically conserved to one or a few isoforms. These mechanisms are produced through the post-translational modification of the regulatory domain independent of Ca^2+^/CaM binding [19]. Erickson et al. showed reactive oxygen species (ROS) as a mechanism of activating CaMKIIδ, through the oxidation of methionine residues (Met281/282) within the regulatory domain. This Met281/282 motif is also found within γ and β isoforms but is changed within the α isoform [20]. Alternatively, the modification of Cys273/290 by nitric oxide induced S-nitrosylation and modulated the activity of CaMKIIδ [21]. O-GlcNAcylation of S280 has also been denoted as a regulatory region of CaMKII leading to pathological sustained kinase activity, leading to cardiac remodeling alongside oxidation of the met281/282 motif [22,23]. As CaMKII within the vasculature remains widely understudied, variation in the basic intrinsic mechanisms of activation between CaMKII isoforms and known splice variants within the variable domain of isoforms is becoming a key area for future research to produce better a understanding of the multifunctional nature of the kinase. 

## 3. Splicing Events in CaMKIIδ and γ Isoforms

Both physiological and pathological functional differences of CaMKII vascular isoforms have been established. However, there is a need for further investigation into the role of these splice variants within vascular CaMKII-mediated signaling pathways. Moreover, there is a need to explore the influence of splice variant ratios in the larger holoenzyme structure and to learn how this impacts cellular functions and signaling pathways. The alternative splicing of CaMKII has been studied largely in cardiac and neurologic contexts. Cardiac-specific approaches tend to focus on the role of δ variants (reviewed by [17,24]), while γ variants are still relatively less studied across all tissue groups. To date, 11 splice variants of the δ isoform [25] have been described; however, the total number of γ variants identified is less clear [26]. While these studies have helped further the understanding of the splice variants, the differential expression of such variants prevents translation to a vascular context [17].

Of the 11 known δ variants, four (δ3, δ1, δ2, and δ9) are expressed within the human myocardium; however, only one (δ2) is regularly discussed within a vascular context [24,27]. δ2 and δ3 variants have been studied widely within a cardiac-specific context. δ3 expression has been shown to increase in cardiac tissues post-myocardial infarction and in heart failure. Similarly, the overexpression of the δ2 variant in a pressure overload-induced mouse model has been shown to augment heart failure [28,29,30]. CaMIKIIδ9 is appreciated as the dominant splice variant in the human heart, with overexpression suppressing Ubiquitin-conjugating enzyme E2T (UBET)-dependent DNA repair, contributing to heart failure and cardiomyopathy [25]. CaMKIIδ1 (also referred to as δA) has high expression in neonates with decreased expression during adulthood, in the presence of upregulation of δ2 and δ3 expression, and δ1 expression is upregulated in chronic heart failure, with hypothesized roles in Ca^2+^ handing through the regulation of L-type calcium channels [26,31]; thus, the distinct roles of these variants in cardiac pathology are clear.

The CaMKII δ3 variant undergoes the translation of exon 14, leading to an 11 amino acid insert within the regulatory domain, rendering the cellular localization of the variant within the nucleus. The δ2 variant, which lacks exon 14 encoding the nuclear translocation signal, is predominantly localized in the cytoplasmic compartment [32]. Thus, the relative amounts of each splice variant in the overall multimeric holoenzyme determine its localization. For example, a higher level of δ2 or δ3 leads to the increased expression of CaMKII in the cytosolic or nuclear compartment, respectively [29,32]. CaMKII δ3 has been shown to increase the transcription of genes related to the hypertrophic growth response of the failing heart. In comparison, CaMKII δ2 is associated with a more severe disease phenotype. This is driven by Ca^2+^-handling dysfunction alongside the upregulation of hypertrophic genes within a much shorter time frame, leading to premature death within in vivo mouse studies [28,30,33,34].

CaMKII δ2 and δ3 are predominantly expressed in cardiac tissue; however, these variants have also been identified in cultured VSMCs from rat aorta. Of these, δ2 remains the more widely discussed vascular splice variant [8,35]. Based on the current literature, the δ2 variant shows pathological VSMC migration and proliferation producing neointimal hyperplasia. However, there is little to no mention of other splice variants, including δ3, negating the potential redundancy or novel functions of δ splice variants in the vasculature [27]. Thus, in light of these pathological mechanisms of the two splice variants, further research of their functions within the vasculature is needed to understand the localization patterns and pathological mechanism of CaMKIIδ variants within heteromeric holoenzymes in vascular disease.

CaMKIIγ splice variants and their specific functionality and cellular localizations remain an understudied area in comparison to δ variants. The distribution of γ splice variants is ubiquitous; however, it is considered the main isoform of vascular tissues under physiological conditions [25]. Four key splice variants have been identified within vascular tissue (γB, γC, γG, and γE); the γB and γC make up majority of the expressed γ gene products. Singer et al. further postulate that γ splice variant expression may have altered regulation throughout development, leading to changes in variant levels throughout such periods [7]. Gangopadhyay et al. noted the presence of CaMKIIγC-1 and γC-2 splice variants within rat aorta cells [16]. The γC-2 variant is different from the γC-1 variant, based solely on a deletion of eight amino acid residues within the motif involved in anchoring ATP in the catalytic domain. This deletion results in low auto-phosphorylation of the γC-2 variant compared with the γC-1; γC-2 is thus less enzymatically active [16].

As differences CaMKII isoforms and splice variants appear to result in unique functional attributes such as different target specificity and regulation mechanisms, current knockout studies, which use non-discriminatory knockout techniques for either total CaMKII or CaMKII isoforms, fail to identify different downstream functions unique to the individual splice variants, as seen with lacking information surrounding CaMKIIγ. Thus, these studies assume that all splice variants conserve the same functions, to the same degree, in spite of their fundamental translational differences. To attenuate this assumption, further research evaluating the contribution of each splice variant to the known physiological and pathological functions of CaMKIIδ and CaMKIIγ in the vasculature is needed.

## 4. The Role of CaMKII in VSMC Migration

The migration of VSMCs is strongly linked to restenosis through neointimal hyperplasia while also being associated with the development of atherosclerosis [36]. The migratory and proliferative characteristics of these cells is intrinsically linked to the cellular phenotype. Under physiological conditions, VSMCs remain in the contractile/differentiated phenotype and maintain vascular tone through intracellular contraction [37]. Following vascular damage, VSMCs de-differentiate to the synthetic phenotype. It is this synthetic phenotype, which is associated with increased VSMC, proliferative and migratory properties promote vascular pathologies [38].

Multiple mechanisms leading to VSMC migration have been identified in recent years. These include the platelet-derived growth factor (PDGF)-stimulated, ROS-stimulated, mitochondrial-translocation and regulation of metalloproteinase-9 (MMP-9), just to name a few [39,40,41,42]. Of these, the platelet-derived growth factor (PDGF)-induced CaMKII activation of VSMC migration is perhaps the most noted mechanism. PDGF is a strong chemoattractant involved in stimulating VSMC migration and hyperplasia during vascular injury [43]. Contractile VSMCs show marked decreases in the activation of CaMKII in response to PDGF, thus leading to a reduced tenancy of migration. In contrast, synthetic VSMCs stimulated with PDGF exhibit increased migration associated with the increase in CaMKII. It is thought that these differences in CaMKII activation in response to PDGF, and subsequent VSMC migration, are linked to intracellular calcium signaling. PDGF induces a rapid increase in intracellular Ca^2+^ within synthetic VSMC, which activates CaMKII and mediates migration. This same response is diminished in contractile VSMC but can be reversed through increases in intracellular calcium [40].

CaMKIIδ is the predominant isoform expressed in the synthetic phenotype. This may suggest a novel regulatory role of this specific isoform and associated splice variants in VSMC migration. Previous studies have shown a reduction in PGDF-induced migration of VSMC with total CaMKII inhibition [40,44]. However, the targeted inhibition of splice variants has produced varying results. Pfleiderer et al. described an increase in VSMC migration with the introduction of kinase-negative CaMKIIδ2, in contrast to the current hypothesis of its role within VSMC migration [44]. Interestingly, Pauly et al. describes the reverse, with reduced VSMC migration in the presence of CaMKII inhibition. However, the attenuated CaMKII isoform within this study is the α isoform [40]. These results are interesting as the α isoform has widely been referred to as a neural-tissue-specific isoform and remains largely understudied within a vascular context [45]. A potential explanation for increased VSMC migration in the presence of attenuated CaMKIIδ2 is the upregulation of a lesser expressed δ isoform, such as δ3. Alternatively, these results may suggest the role of other regulatory mechanisms that function independently of CaMKII. PDGF-induced activation has been described as an indirect mechanism in which PDGF induces ERK1/2 activity, which in turn activates CaMKII; while true, it is also noted that ERK1/2 also regulates migration independently of CaMKII activation [46,47]. This may be an explanation for enhanced VSMC migration in spite of CaMKIIδ2 knockout. However, such hypotheses still remain largely unproven.

**Figure 1:**
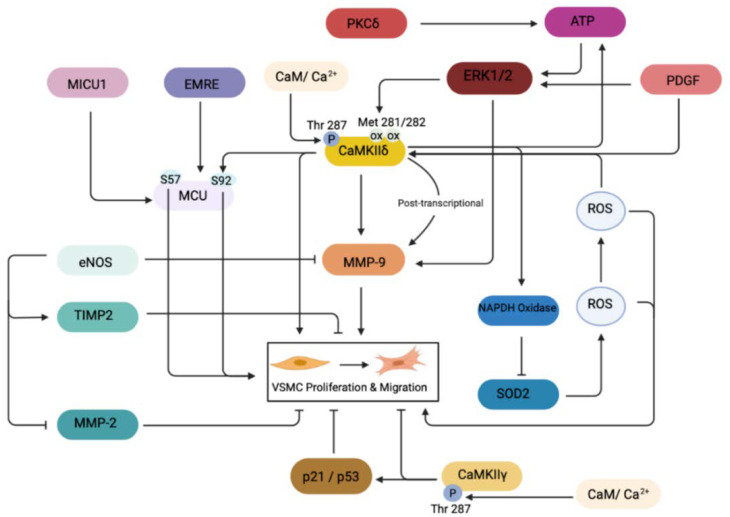
The regulatory mechanisms of vascular smooth muscle cell migration and proliferation. Potential up- and down-regulatory mechanisms of vascular smooth muscle cell migration (VSMC) involving both CaMKII-dependent and -independent mechanisms at both transcriptional and post-transcriptional levels. CaMKIIγ expression and activation leads to promotion of VSMC proliferation and migration, both directly and indirectly, through p21 and p53 cell cycle inhibitors. CaMKIIδ expression leads to increased VSMC migration and proliferation. PKCδ and PDGF both upregulate CaMKIIδ to increase VSMC migration and proliferation. PKCδ works by upregulating ERK1/2 activity via ATP, leading to increased CaMKIIδ activity. PDGF acts directly and indirectly via ERK1/2 to upregulate CaMKIIδ, leading to VSMC migration and proliferation. MCU expression is upregulated by MICU1 and EMRE, leading to the promotion of VMSC migration and proliferation. MMP-9 is upregulated by CaMKIIδ and ERK1/2, leading to increased VSMC migration and proliferation, while eNOS downregulates MMP-9. eNOS further downregulates MMP-2 and upregulates the tissue inhibitor of metalloproteinases 2 (TIMP2) to decrease VSMC migration and proliferation.

The ROS-dependent activation of CaMKII leading to VSMC migration is an important mechanism stimulating migration (Figure 1). For example, VSMCs become activated due to increased ROS levels following vascular injury [48]. The ROS-dependent activation of CaMKII has been demonstrated through knockout models. The mutation of methionine residues 281/282 in CaMKIIδ prevented the oxidation-dependent activation of CaMKII, thus decreasing VSMC migration in such models [49]. VSMC migration through the ROS-CaMKIIδ pathway has been shown to depend on the activation of matrix metalloproteinases such as MMP-9 [41]. MMP-9 promotes VSMC migration during states of vascular injury, leading to neointimal hyperplasia [50]. Matrix metalloproteinases are linked to the degradation of the extracellular matrix (ECM) and basement membrane within the arterial wall, facilitating VSMC migration [36,51]. The activation of MMP-9 is complex and influenced by a multitude of ROS pathways. Scott et al. suggests CaMKIIδ as a downstream mediator of MMP-9 because its expression decreases in CaMKIIδ^−/−^ knockdown models via a loss of CaMKII-mediated stabilization of MMP-9 mRNA [36,46]. These findings link the δ isoform to VSMC migration, potentially through the Met281/282 motif. Interestingly, this motif is conserved in the γ and β isoforms too. All CaMKII isoforms contain a single cysteine in the regulatory domain, which aids in activation; however, this is insufficient to cause activation in CaMKIIδ, thus rendering the Met281/282 more important in oxidation dependent activation mechanisms [20]. However, as discussed earlier, there are several variants of CaMKIIδ, but, to date, studies have only focused on the δ2 isoform. It is possible that other δ variants may also promote VSMC migration. For example, the deletion of the oxidation motifs in CaMKIIδ2 led to a compensatory activation of CaMKII activity and therefore no attenuation of VSMC migration [49]. It is unlikely this compensation is due to other isoforms, such as γ, because this isoform is associated much more closely with the contractile VSMC phenotype. CaMKIIγ has also been shown to reduce VSMC proliferation and migration by increasing the expression of p53 and p21 (cell cycle inhibitors) and CaMKIIδ to promote such proliferation [35]. Thus, evaluating the roles of δ isoform splice variants in this compensatory effect may further delineate the ROS-induced CaMKII activation of VSMC migration.

The ROS-induced activation of CaMKII is primarily through a nicotinamide adenine dinucleotide phosphate (NADPH) oxidase-dependent mechanism [20,52]. However, activated CaMKII then regulates NADPH oxidase in what appears to be a regulatory loop. For instance, knockdown models of CaMKIIδ oxidation through the mutation of the Met281/282 motif have demonstrated decreased NAPDH-oxidase expression and increased expression of superoxide dismutase (SOD2) [49]. Mitochondria produce the key ROS, superoxide; however, they are protected from oxidative stress through the expression of SOD2 [53]. SOD2 inhibits the angiotensin-II-induced migration of VSMC, which is associated with superoxide production through NADPH-oxidase [22]. The findings of both Nishio et al. and Zhu et al. link CaMKII as a regulator of SOD2 and NADPH-oxidase expression; increased activity of SOD2 within the mitochondria thus reduces ROS production and results in decreased VSMC migration [49,52]. It follows that different splice variants of the δ isoform may act at the different levels of the pathway, but such a hypothesis is yet to be investigated.

Mitochondrial Ca^2+^ uniporter (MCU) is the main controller of Ca^2+^ influx into the mitochondria. CaMKII has been linked as a regulator of MCU, controlling Ca^2+^ influx in the mitochondria of VSMC to allow for MCU-dependent VSMC migration during vascular pathologies such as neointimal hyperplasia. This control of MCU-dependent Ca^2+^ influx by mitochondrial CaMKII is needed for mitochondrial translocation to the leading edge in migratory VSMCs, where they aid in cytoskeletal remodeling, thus noting a key function of the kinase [54]. Similar trends are seen across different cell lines, highlighting the function of MCU in VSMC migration [54,55]. The phosphorylation of S92 is key for regulating the activity of MCU, and the inhibition of CaMKII decreases phosphorylation events at this site linking the kinase to MCU Ca^2+^ influx; however, other sites such as S57 have also been demonstrated as alternative phosphorylation sites. The MCU is also regulated by the activation of mitochondrial calcium uptake 1 (MICU1) and essential MCU regulator (EMRE), both of which are mitochondrial proteins forming subunits of the uniporter, alongside the MCU protein. It is not yet known if mitochondrial CaMKII is involved in these alternative regulatory mechanisms [56,57]. Nguyen et al. also describe decreased mitochondrial ROS production with the inhibition of mitochondrial CaMKII due to the requirement of upstream Ca^2+^ influx in producing ROS, thus further suggesting the role of CaMKII as an upstream regulator [54]. The splice variant specificity of MCU activation still remains unknown; however, due to the relative expression levels and functions identified in previous studies, it is likely that activation is related to the δ isoform.

The complexity of VSMC migration within vascular injury and its link to CaMKII, in particular CaMKIIδ, cannot be understated. However, based on the information known regarding the activation and cellular localization of isoforms and their splice variants, there is an increasing need for more precise research on the role of different splice variants in VSMC migration [44]. While a range of genetic and pharmacological knockdown models of CaMKII have been shown to decrease migration, they attenuate any novel functions of splice variants within these mechanisms due to a lack of specificity.

## 5. Reciprocal Expression of CaMKII Isoforms in VSMC Phenotypic Switching

**Figure 2:**
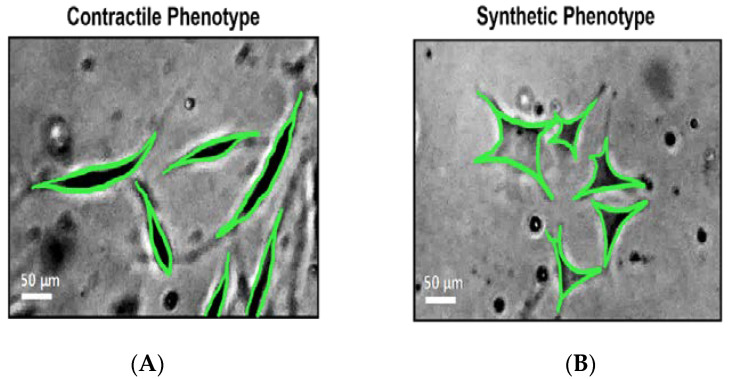
Phenotypic switch of human coronary artery smooth muscle cells (HCASMCs). Atherosclerotic-mimic conditions (150 µM H_2_O_2_ and 40 ng/ mL TNF-α) trigger a phenotypic shift from the contractile to synthetic form: (**A**) representative photograph of HCASMCs under control conditions, exhibiting a classical contractile, elongated shape; (**B**) representative photograph of HCASMCs under atherosclerotic conditions, exhibiting a classical synthetic, rhomboid shape. Scale bar represents 50 µm.

Throughout development, VSMCs will reach a differentiated (contractile) phenotype associated with contractility and cell cycle regeneration (Figure 2A). However, this differentiated state is not terminal as VSMCs can convert to their de-differentiated states in periods of angiogenesis and repair following injury [38] (Figure 2B). This de-differentiated state, also known as the synthetic phenotype, is associated with proliferation, migration, and the expression of gene products that drive these events. CaMKII isoform expression has been shown to modulate the transition between different VSMC phenotypic states [6]. CaMKIIδ is strongly associated with VSMC migration, denoting it as the predominate isoform within synthetic cells, while CaMKIIγ is related to the contractile functions of the differentiated phenotype [3,58].

The opposing functions of the two isoforms within the different phenotypes of VSMCs has been described in vivo, with CaMKIIγ reducing proliferation through increased expression of p53 and p21 (cell cycle inhibitors) and CaMKIIδ promoting such proliferation [35]. CaMKIIδ-mediated VSMC proliferation signaling pathways, similarly to VSMC migration, are complex. The inhibition of p21 and the upregulation of Mdm2 (a p53 E3 ligase) by CaMKIIδ led to the proliferation of VSMCs [4]. Growth factors such as TNFα, PDGF, and FBS are also linked to CaMKII-mediated VSMC proliferation (as reviewed in [1]). ERK has also be identified as a promoter of VSMC proliferation through the CaMKII-dependent α-adrenergic activation of ERK. [59]. CaMKIIγ, instead, is associated with the contractile VSMC phenotype leading to VSMC contraction through two mechanisms: Ca^2+^ influx, and its interaction with contractile proteins [59,60]. The contraction of VSMC is driven by intracellular Ca^2+^ transients. These transients are mediated through L-type high voltage-gated Cav1.2 calcium channels, which have been shown to be phosphorylated by CaMKII in VSMCs [60,61]. While these studies do not specify the specific CaMKII responsible for this phosphorylation, other studies have associated the γ isoform of CaMKII with contractile proteins. The inhibition of CaMKIIγ reduces myosin light-chain (MLCK) activity in tonic force maintenance and the subsequent myosin light-chain (LC20) phosphorylation required for contractile functions [58]. More specifically, the CaMKIIγG-2 splice variant been linked to VSMC contraction, with binding specificity to the VSMC cytoskeleton. When activated, it causes the phosphorylation of vimentin, which is released into the cytosol to target cortical dense plaques. This cascade is responsible for activating ERK1 and triggering LC20 phosphorylation and thus VSMC contraction [12]. While this pathway marks the link between the γ isoform and VSMC contractility, there are numerus other redundant mechanisms that regulate this contractility, such as LC20 phosphorylation directly by Ca^2+^/calmodulin and MLCK [62].

Phenotypic switching from the contractile VSMCs to synthetic cells is seen with reciprocal up- and downregulation of CaMKIIδ and CaMKIIγ expression, respectively [27]. This phenotypic switch is seen in response to stimuli such as growth factors, cytokines, and mechanical influences, which alter gene transcription, and up- or downregulating proteins associated with the two VSMC phenotypes. This alteration in gene transcription is associated with a different Ca^2+^ signaling pathway. Cells expressing the synthetic phenotype show a short-branched structure compared to the more elongated phenotype of differentiated VSMC (Figure 2A,B), with the downregulation of RyR, KCa1.1 and L-type Ca^2+^ channels and upregulation of TRPC and KCa3.1 Ca^2+^ channels [60,63]. The current literature suggests that these changes in Ca^2+^ handling may be related to the differential expression of the δ and γ CaMKII isoforms. Saddouk et al. first noted the functions of CaMKIIγ within the contractile VSMC phenotype with the subsequent downregulation of the γC splice variant and the concomitant upregulation of CaMKIIδ2 following vascular injury and associated the CaMKIIγ downregulation and CaMKIIδ upregulation with the promotion of the synthetic phenotype [35].

## 6. Regulation of CaMKIIγ and CaMKIIδ in VSMCs

An understanding of how the expression pattern of CaMKII δ and γ are regulated during VSMC phenotype switching is emerging [64,65]. The prevailing mechanism for CaMKIIγ expression during the phenotypic switch is suggested to be regulated by the methylation and demethylation patterns of the γ isoform promoter. Within the contractile phenotype, the promoter is demethylated, whereas the synthetic phenotype displays methylation. The regulatory mechanisms of this phenotype switch, however, still remain in their infancy, opening a new area of research to produce a better understanding of CaMKII splice variants in VSMC migration seen in vascular disease states [64].

Previous studies have described ten-eleven translocation-2 (TET2) as a master regulator of the VSMC differentiated phenotype [66]. TET2 is one of three enzymes within the TET family [65]. These enzymes oxidize 5-methylcytosine (5mC) to 5-hydroxymethylcytosine (5hmC), which is then converted to unmethylated cytosine-producing DNA demethylation and the alteration of gene expression [67]. High levels of TET2 are seen within the differentiated phenotype of VSMCs, but TET2 and 5hmC are reduced in the synthetic phenotype of VSMCs [64,66]. The *Camk2g* promoter is demethylated in differentiated VSMCs [66]. However, following vascular injury this promoter undergoes methylation, leading to the decreased expression of CaMKIIγ, associated with the de-differentiation of VSMCs to the synthetic phenotype [27]. The regulation of this phenotypic switch has been suggested to be associated with the promotion of thymine DNA glycosylase (TDG) mRNA expression and subsequent protein expression both in vitro and in vivo. Following the silencing of the TDG gene through cytosine methylation/demethylation patterns, the expression of CaMKIIγ was reduced, thus linking TDG to the synthetic phenotype and making it a potential regulator of the phenotypic switch seen post-vascular injury [64]. The role of TDG and methylation patterns on regulation are still relatively poorly understood. Liu et al. noted decreased levels of both CaMKIIγC and γB splice variants following changes in methylation patterns, re-enforcing the role of methylation as a key regulator of this phenotypic switch [64].

The CaMKII-dependent regulatory mechanisms of VSMC phenotype plasticity do not appear to be the only regulatory mechanisms. Myocardin (MYOCD) and serum response factor (SRF) are known to induce the contractile phenotype while Kruppel-like factor 4 (KLF4) has been linked to the synthetic phenotype [66,68,69]. TET2 has been suggested as an upstream regulator of both SRF and MYOCD, thus suggesting the regulation of VSMC phenotypic proteins independent of CaMKIIγ expression [66]. While a role for the methylation/demethylation of the promoter is an important regulator of CaMKIIγ expression, it also influences the regulation of other VSMC proteins associated with synthetic and contractile phenotypes, highlighting the complexity of this regulatory mechanism [70].

While important in CaMKIIγ regulation, promoter methylation and demethylation patterns play a lesser role in the regulation of CaMKIIδ expression. Liu et al. noted that the Camk2d promoter was demethylated within the synthetic VSMC phenotype; however, the expression of CaMKIIδ was not significantly altered with changes in methylation patterns, suggesting this is not the predominant regulatory mechanism of the δ isoform [64]. Instead, it is thought that the expression of CaMKIIδ is instead regulated through mircoRNAs (miRs), specifically miR-30 family members, such as miR-30, miR-143, and miR-145. The regulatory role of these miRNAs’ CaMKIIδ expression increases with the down-regulation of miR-143 and miR-145 expression, which is seen largely in the synthetic phenotype of VSMC. As CaMKIIδ expression shows a marked increase during the phenotypic switch, it supports miRNAs as a key inhibitor in this regulatory mechanism [45]. The expression of miR-145 is upregulated during the phenotypic switch from the synthetic to contractile VSMC phenotype, while key proteins (KLF4) of the synthetic phenotype are suppressed [69,71]. miR-145 regulates this phenotypic switch by both directly targeting CaMKIIδ expression and indirectly through the modification of key proteins such as KLF4; however, the predominant mechanism is yet to be proven [45]. In cardiomyocytes, miR-145 expression reduces ROS production, decreasing the ROS-induced expression of CaMKIIδ as well as directly attenuating CaMKIIδ expression [72]. While this is yet to be shown within VSMC, it suggests the complex involvement of miRNAs in the regulation of CaMKIIδ. Interestingly, miR-145 is abundant within arteries and VSMCs, highlighting the need for more research to confirm these findings in VSMC to better understand the regulatory role of the miR [27,72].

Micro-RNAs have also been shown to regulate CaMKIIγ expression; however, this is yet to be seen in VSMC. miR-219 overexpression reportedly downregulates the expression of CaMKIIγ to reduce Ca^2+^ influx through NMDA-R signaling in psychiatric disorders [73,74,75,76]. Methylation patterns in the promoter of miR-219 have since been linked to its expression pattern. The hypermethylation of the miR-219 promoter is seen in conditions such as Complete Freund’s Adjuvant (CFA)-induced chronic inflammation pain, leading to reduced expression of the miR. The demethylation of the promoter increases miR-219 expression and subsequently reduces CaMKIIγ levels [77]. Thus, if miR-219 functions similarly in VSMC, it would suggest increased expression of miR-219 within the synthetic phenotype; however, this is yet to be proven. As the CaMKIIγ promoter is also regulated by methylation, these findings may suggest a more complex regulatory mechanism. Alternatively, the methylation/demethylation regulation of the kinase, and the miRNA that regulate it, may be linked to the tissue-specific expression of the different isoforms [64].

These findings highlight the complexity of CaMKIIδ and CaMKIIγ regulation during the phenotypic switch of VSMCs. The methylation patterns of the miR-219 promoter and CaMKIIγ promoter play a large role in the regulation of CaMKIIγ. This is seen to a far lesser extent in CaMKIIδ. Although the *Camk2d* promoter is demethylated in the synthetic phenotype of VSMC, this does not influence its expression significantly. Instead, it is regulated through miRNA-30s, which appear to alter its expression. Due to the conserved miR-30 binding region within the *Camk2d* promoter, it is likely that regulation by miRNAs does not differ largely between splice variants of CaMKIIδ; however, this too is yet to be shown [73]. 

## 7. Role of CaMKII in VSMC Hypertrophy

**Figure 3:**
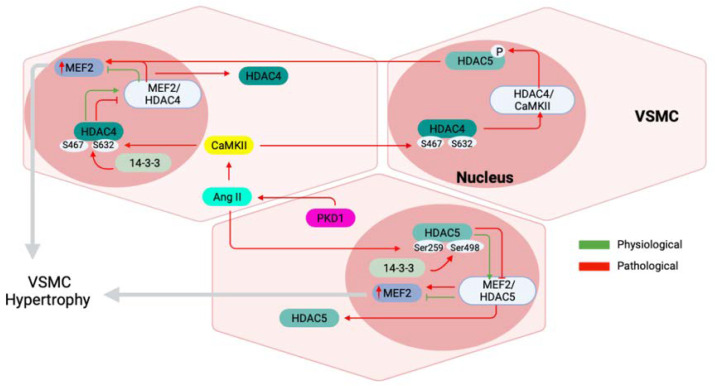
Pathological mechanisms of VSMC hypertrophy. Angiotensin II (AngII) leads to phosphorylation of HDAC4/5 chaperone 14-3-3 binding sites through CaMKII-dependent (HDAC4) and CaMKII-independent (HDAC5) mechanisms. Under physiological conditions, HDAC4/5 forms complexes with myocyte enhancer factor-2 (MEF2). Under pathological conditions, complex formation is downregulated and MEF2 leads to activation of hypertrophic genes for contractile proteins such as beta-MHC, atrial natriuretic factor (ANF), and transcription factor KLF5, leading to VSMC hypertrophy.

Hypertension is caused by the stiffening of artery walls, leading to decreased regulation of blood pressure while increasing atherosclerotic risk [78]. VSMC hypertrophy contributes to worsening hypertension [36,79]. CaMKII has been implicated in VSMC hypertrophy through the angiotensin II (ANG II)/CaMKII mediated pathway [80] (Figure 3). This regulatory mechanism was first described in cardiomyocytes, where ANG II activates CaMKII, which, in turn, phosphorylates type II histone deacetylases (HDACs) [80,81]. HDACs are transcription repressors of muscle enhancer factor 2 (MEF2) [82]. MEF2 is a family of transcription factors, with both cardiac and vascular isoforms that regulate genes associated with pathological hypertrophy [80].

Type II HDACs include HDAC4, −5, −7, and −9; however, the ANG II-CaMKII cascade appears to phosphorylate HDAC4 to activate MEF2 [80,82]. In order for this activation to occur, HDACs are transported to the nucleus following chaperone (14-3-3) binding; docking sites for these chaperones are exposed following the CaMKII phosphorylation of HDAC4 at S467 and S632. Evidence suggests this docking site is specific to HDAC4 and thus suggests a more specific role of type II HDACs [33]. The inhibition of CaMKII within this cascade leads to reduced cardiomyocyte and VSMC hypertrophy, both in vitro and in vivo, through decreased MEF2 activation. Complete CaMKII knockout models have been used to produce these findings; however, CaMKIIδ2 has been suggested to play a larger role in studies using CaMKIIδ overexpression, with HDAC4 phosphorylation by CaMKIIδ2 producing increased retention in the cytoplasm and the downstream hypertrophic response [83].

HDAC5, following CaMKII-independent phosphorylation, can produce ANG II-induced activity of MEF2, resulting in VSMC hypertrophy [84]. While this activity is independent of CaMKII, HDAC5-MEF2 activation has since become linked to the kinase through a regulatory function of HDAC4 upstream [85]. Prasad et al. showed increased expression of genes controlling ECM composition, leading to reduced elasticity and subsequent arterial stiffening within a CaMKII-knockout model. Although the exact role of CaMKII remains undetermined, CaMKIIδ3 and CaMKIIδ2 have both been reported to increase the transcription of genes related to the hypertrophic response in cardiomyocytes [78]. The function of these splice variants in cardiac tissue suggests the need for more vascular specific splice variant studies related to VSMC hypertrophy to understand the vascular hypertrophic events [28,30,33,34].

The evidence has begun to support a much wider role of the CaMKII in hypertension, beyond VSMC hypertrophy, such as through the phosphorylation of MLCK leading to increased tonic force maintenance [62]. Alternatively, the phosphorylation of PLA2 by CaMKII leads to increased arachidonic acid release and metabolism, elevating levels of 20-hydroxeicosatetraenoic acid, which increases vasoconstriction and VSMC proliferation [86]. These studies show the multifaceted roles of the kinase in contributing to VSMC hypertrophy and subsequent hypertension. What is yet to be identified is the contribution of specific isoform/variants to this process. CaMKIIδ has a role to play in VSMC hypertrophy at multiple levels, with δ2 and δ3 splice variants playing key roles in this pathogenesis. This area still remains in its infancy and more research is needed to confirm the roles of different splice variants and isoforms in this mechanism. 

## 8. Conclusions

Although there has been a large basis of research produced evaluating the role of CaMKII and its isoforms, in the pathophysiology of vascular conditions, far less is understood about the contribution of its individual isoform splice variants. Nearly all research produced within the vascular context is done through complete knockout models of CaMKII, masking any functional differences between spliced products across the four isoform genes. Current research suggests opposing functions of the two vascular isoforms (δ and γ) in VSMCs with reciprocal expression during pathological and physiological states, respectively. CaMKIIδ appears at multiple levels as both a regulator and mediator of VSMC migration, indicating the importance of ROS production during the injury state as one mechanism of activation alongside numerous others, suggesting potential redundancy in therapeutic agents limited to a single pathway. Perhaps more importantly, CaMKIIγ plays a protective role in the vasculature, with downregulation leading to phenotypic switch. The ubiquitous distribution, complexity of function, regulation, and localization of isoforms across tissues indicates that therapies targeted at isoforms or splice variants are needed to retain protective functions of the kinase while minimizing the vascular-specific deleterious effects seen in vascular disease states. Further to this, research on CaMKII variants in the vasculature is complexly intertwined with its role in cardiac tissue and pathology, suggesting the further need for the evaluation of splice variants across the two tissues. Future research requires an in depth look into the functions of the vascular splice variants and how they regulate pathological functions of VSMCs in disease states such as atherosclerosis and hypertension.

## Data Availability

Not applicable.
